# Detection of Plasma Protease Activity Using Microsphere-Cytometry Assays with *E*. *coli* Derived Substrates: VWF Proteolysis by ADAMTS13

**DOI:** 10.1371/journal.pone.0126556

**Published:** 2015-05-18

**Authors:** Shobhit Gogia, Chi Y. Lo, Sriram Neelamegham

**Affiliations:** Department of Chemical and Biological Engineering and NY State Center for Excellence in Bioinformatics and Life Sciences, State University of New York, Buffalo, New York, United States of America; Michigan State University, UNITED STATES

## Abstract

Protease levels in human blood are often prognostic indicators of inflammatory, thrombotic or oncogenic disorders. The measurement of such enzyme activities in substrate-based assays is complicated due to the low prevalence of these enzymes and steric hindrance of the substrates by the more abundant blood proteins. To address these limitations, we developed a molecular construct that is suitable for microsphere-cytometer based assays in the milieu of human blood plasma. In this proof of principle study, we demonstrate the utility of this substrate to measure metalloprotease ADAMTS13 activity. The substrate, expressed in *E*. *coli* as a fusion protein, contains the partial A2-domain of von Willebrand factor (VWF amino acids 1594–1670) that is mutated to include a single primary amine at the N-terminus and free cysteines at the C-terminus. N-terminus fluorescence conjugation was possible using NHS (N-hydroxysuccinimide) chemistry. Maleimide-PEG(Polyethylene glycol)_n_-biotin coupling at the C-terminus allowed biotinylation with variable PEG spacer lengths. Once bound to streptavidin-bearing microspheres, the substrate fluorescence signal decreased in proportion with ADAMTS13 concentration. Whereas recombinant ADAMTS13 activity could be quantified using substrates with all PEG repeat-lengths, only the construct with the longer 77 PEG-unit could quantify proteolysis in blood plasma. Using this longer substrate, plasma ADAMTS13 down to 5% of normal levels could be detected within 30 min. Such measurements could also be readily performed under conditions resembling hyperbilirubinemia. Enzyme catalytic activity was tuned by varying buffer calcium, with lower divalent ion concentrations enhancing cleavage. Overall, the study highlights the substrate design features important for the creation of efficient proteolysis assays in the setting of human plasma. In particular, it emphasizes the need to introduce PEG spacers in plasma-based experiments, a design attribute commonly ignored in immobilized peptide-substrate assays.

## Introduction

The total protein concentration of human blood plasma is 60–80mg/mL with serum albumin, globulins, transferrin, fibrinogen and a handful of additional molecules constituting ~99% of the total content [1,2]. A much smaller portion of this complex mixture (<1%) is composed of other proteins including, but not limited to, the coagulation factors and blood proteases. While the overall concentration of these proteolytic enzymes is small, they still have a profound effect on diverse biologically significant processes like thrombosis, inflammation and cancer metastasis. For example, ADAMTS13 (a disintegrin and metalloprotease with thrombospondin type-1 motif 13) is a constitutively active blood metalloprotease that cleaves ultralarge von Willebrand factor (VWF) in circulation to produce VWF units with smaller molecular mass [3,4]. The absence of this protease prevents the breakdown of VWF and this contributes to life-threatening thrombosis in microvessels, a disorder called thrombotic thrombocytopenic purpura (TTP). Additionally, thrombin generation and fibrin gel formation during secondary hemostasis is regulated by a number of blood coagulation factors, most of them being serine proteases that act by cleaving downstream proteins. Other human proteases found in blood including kallikreins, metalloproteases and cathepsins are also used as prognostic indicators of various diseases [5–7].

From the diagnostics perspective, it would be attractive to create multiplex technologies that can measure diverse proteolytic enzyme activities in human blood rapidly at low cost, particularly if this can be done in small volumes in the context of point-of-care testing. Flow cytometry based microsphere methods are an attractive choice for such assays since the loss of microsphere-associated fluorescence upon proteolysis can be readily measured, and this provides a straightforward readout of proteolysis rates [8]. Additionally, microfluidics based devices are starting to be developed with flow cytometry capabilities [9], and the integration of both these platforms appears to be within reach if critical bottlenecks are resolved. In this regard, previous studies have utilized ‘microsphere-cytometry’ based approaches to monitor the proteolytic activity of enzymes in pure form for the purpose of high-throughput molecular screening and drug discovery [8,10]. The current manuscript presents an extension of this approach for the analysis of proteolytic activity in complex mixtures, specifically in the milieu of blood plasma. It describes a strategy for the expression of substrates of human proteolytic enzymes in *E*. *coli*, and methods to functionalize them for diagnostic applications. The long-term goal is to extend this approach to point-of-care testing using microfluidics technology.

As proof of principle, the current manuscript describes a workflow for the creation of microsphere-cytometry substrates in *E*. *coli* for the detection of ADAMTS13 proteolytic activity. We chose to assay this enzyme activity since the interaction interface between VWF and ADAMTS13 is more complex and extensive compared to other proteases in the blood coagulation cascade [11,12]. In this regard, while ADAMTS13 specifically cleaves the Y1605-M1606 scissile bond that is buried within the A2-domain of VWF, the minimal VWF substrate required to assay this enzyme activity includes 73 amino-acids stretching from D1596 to R1668 [11,13], with 77 amino acid peptides also being used in some applications [14]. Such a large substrate is necessary since ADAMTS13 must simultaneously recognize several remote VWF exosites before the scissile bond can be cleaved. Thus, if a substrate for complex enzymes like ADAMTS13 can be established, substrates for additional enzymes could be developed on this backbone. Specific design considerations while developing the new ADAMTS13 substrate included: a. The ability to produce substrates in *E*. *coli* and functionalize them in stable form since such a platform can enable rapid substrate prototyping and scale-up. b. The measurement of low levels (<10% normal levels) of ADAMTS13 activity in human plasma since such sensitivity is necessary for the clinical diagnosis of TTP [4]. During this pathology, the reduction of ADAMTS13 activity in blood enhances VWF multimer size in circulation, and this causes the formation of platelet-rich microthrombi in the small vessels of multiple organs. c. The ability to accurately measure enzyme activity in the presence of bilirubin. In this regard, a variety of clinical studies have documented the wide prevalence of hyperbilirubinemia in TTP patients [15–17], and severe hyperbilirubinemia and thrombocytopenia has also been noted secondary to TTP in newborns [18]. Fluorogenic substrates that measure ADAMTS13 activity often fail in the presence of bilirubin due to overlapping absorbance/fluorescence spectra [19], and microsphere assays may be designed to overcome this limitation.

## Materials and Methods

### ADAMTS13

All human subject protocols were approved by the University at Buffalo Institutional Review Board (IRB). Written consent was obtained from these participants based on these approved protocols. Human recombinant ADAMTS13 (rADAMTS13) and platelet poor plasma (PPP) were the source of ADAMTS13 activity. Platelet poor plasma (PPP) was separated from blood drawn from healthy non-smoking volunteers in either 50U/ml heparin, 0.4% w/v sodium citrate or 0.4% w/v citrate-dextrose solution [20]. The PPP obtained in this manner is referred to as ‘plasma’ in the manuscript. Heat inactivated plasma (HIP) was prepared by heating plasma at 56°C for 30 min. rADAMTS13 was prepared as described previously [14]. The activity of this protein was defined by calibrating the reagent against pooled plasma drawn from 4 normal human donors (ADAMTS13 activity of this pooled plasma was set to 1U/ml). Heat inactivation of rADAMTS13 was performed by denaturing enzyme at 95°C for 60min.

### ADAMTS13 substrate expression in E. coli

**Fig 1:**
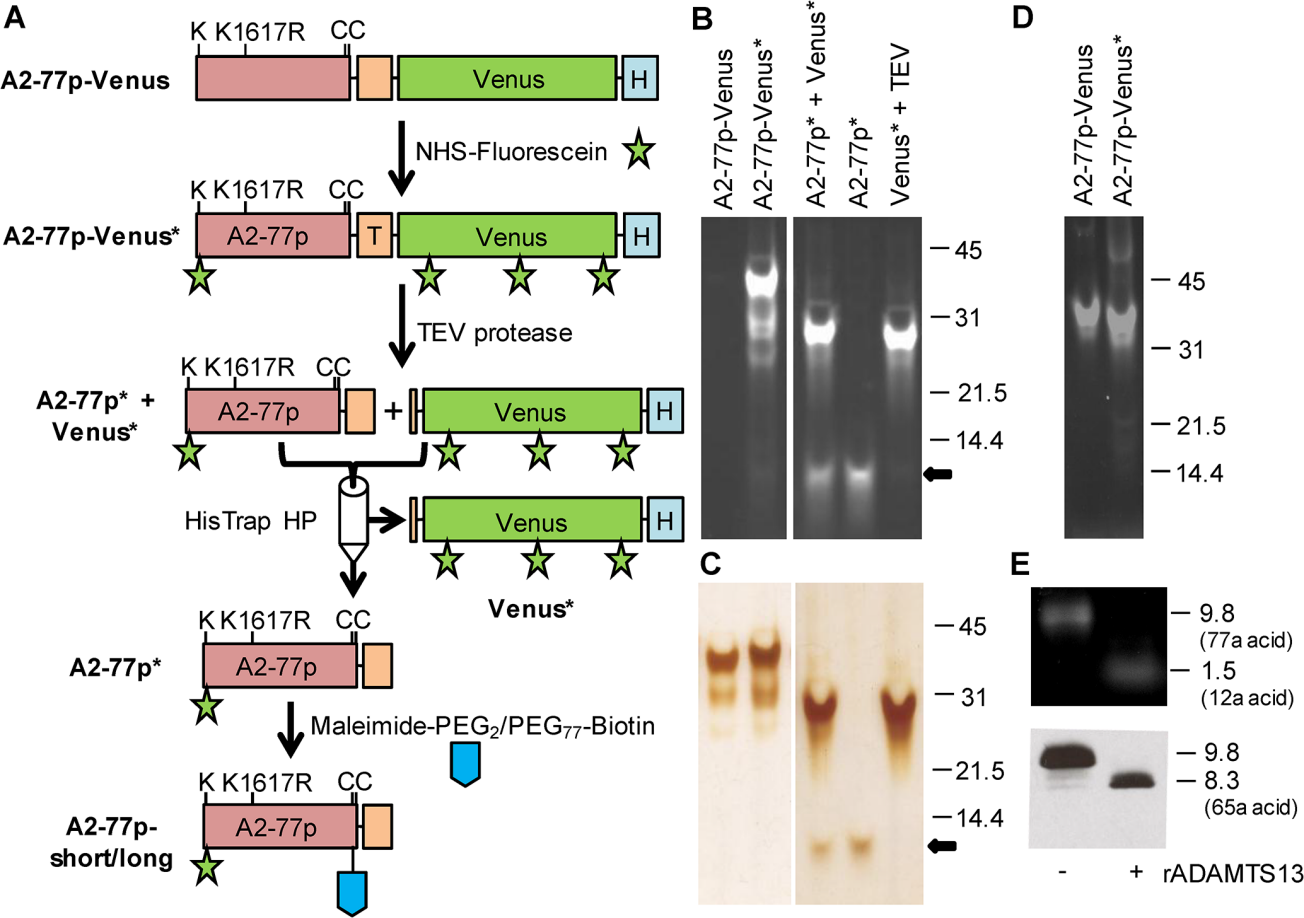
ADAMTS13 substrate synthesis. **A.** The 37.3 kDa fusion protein A2-77p-Venus, purified from *E*. *coli*, was labeled with fluorescein yielding A2-77p-Venus*. A2-77p-Venus* incubated with TEV protease resulted in two fragments A2-77p* and Venus*, which were separated using a HisTrap HP column. A2-77p* was coupled with a biotinylation linker that had either 2 or 77 PEG spacer units. The final product that is coupled to both fluorescein and biotin is called A2-77p-short (2 PEG units) or A2-77p-long (77 PEG units). A similar protocol was applied to create negative control proteins where residues Y1606 and M1605 were replaced by alanine. T—TEV cleavage site, H—7x His tag. **B.** Starting material and intermediates resolved using a 4–20% gradient gel under reducing conditions. Fluorescence is due to fluorescein alone since Venus signal is lost upon boiling. **C.** Silver stain of gel in panel B. Peptide of interest (A2-77p*) appears at 9.8 kDa (arrow). **D.** A2-77p-Venus (unlabeled) and A2-77p-Venus* (fluorescein labeled) resolved using a 4–20% gradient gel under non-reducing conditions. Fluorescence in the gel is due to both Venus and fluorescein. **E.** A2-77p-short was incubated with or without 4.4U/ml rADAMTS13 for 90min. Product was resolved using a tricine gel. Top panel shows fluorescence under UV illumination. Bottom panel shows western blot of the gel using anti-biotin antibody. As expected, the 9.8 kDa substrate is cleaved into 1.5 kDa fluorescent (top gel) and 8.3 kDa biotinylated product (bottom gel).

Polymerase chain reaction (PCR) primers used for molecular biology are listed in S1 Table. pcDNA3.1 plasmid containing full length VWF, and pRSETB plasmids encoding for FRET proteins L-VWF, XS-VWF (deposited at Addgene) and XS-VWF(AA) were available from previous studies [14]. In L-VWF and XS-VWF, the full length VWF-A2 or just the 77 amino-acid truncated A2-peptide (amino acid 1594–1670) are flanked by the CFP variant Cerulean (donor) and YFP variant Venus (acceptor). XS-VWF(AA) is identical to XS-VWF, only the Y1605 and M1606 residues were mutated to alanine. To create DNA encoding for A2-77p-TEV-His, forward primer NdeI1594Lys and reverse primer VWFK1617R were applied on the VWF template in pcDNA3.1 to generate a megaprimer. This PCR step mutated Lys to Arg at 1617 of VWF (K1617R), and introduced an NdeI cleavage site and new Lys (K) residue before Gln 1594 (Fig 1A). This mega primer was used as a forward primer along with the BstBI1670 reverse primer on the same VWF template to create a fragment with NdeI and BstBI overhangs. This DNA fragment was ligated into the pRSETB vector containing L-VWF digested with NdeI and BstBI, to replace the FRET construct Venus-A2-Cerulean [14]. The resultant product is termed ‘A2-77p-TEV-His’. For creating the negative control (‘A2-77p(AA)-TEV-His’), the same series of reactions was performed except that the first PCR step was performed using XS-VWF(AA) [14], instead of VWF, as template. To create the fusion proteins A2-77p-Venus and A2-77p(AA)-Venus, forward primer BstBIVenTEV and reverse primer HindIIIVenHis were used on Venus-GalT (Addgene # 11931, Cambridge, MA) to amplify Venus with the TEV cleavage site at N-terminus and 7×His-tag at the C-terminus. This product was ligated into A2-77p-TEV-His and A2-77p(AA)-TEV-His (replacing original TEV-His fragment) to give ‘A2-77p-Venus’ (plasmid deposited at Addgene) and ‘A2-77p(AA)-Venus’, respectively. In both constructs, Venus is preceded by a TEV cleavage site and it is followed by a His-tag. Both fusion proteins, A2-77p-Venus and A2-77p(AA)-Venus, were expressed in *E*. *coli* strain BL21 Star (DE3) and protein product was purified as detailed in S1 Methods.

### Substrate conjugation

To label A2-77p-Venus and A2-77p(AA)-Venus with NHS-fluorescein (Thermo-Pierce, Rockford, IL, USA), 15-fold molar excess NHS-fluorescein was added to 5mg/mL protein for 1h at room temperature in phosphate buffered saline (PBS) at pH 8.0. The reaction was quenched using excess 1M Tris, and labeled protein was separated from unreacted NHS-fluorescein using a P-30 fine gel resin column (Bio-Rad Laboratories, Richmond, CA, USA) (Fig 1A). The resultant protein is called A2-77p-Venus* (* denotes fluorescein) and A2-77p(AA)-Venus*. Next, TEV protease produced in BL21 (DE3)-RIL (see S1 Methods) was added to A2-77p-Venus*/A2-77p(AA)-Venus* (2μg TEV/ 100μg protein) in the cleavage buffer (50mM Tris, pH 8.0, 0.5mM EDTA, 1mM DTT) overnight at 20°C. The mixture was then passed through a HisTrap HP column (GE Healthcare, Piscataway, NJ, USA) equilibrated with 20mM HEPES (pH 7.4) containing 300mM NaCl. The flow-through containing the fluorescein conjugated peptide A2-77p*/A2-77p(AA)* was collected, concentrated and buffer-exchanged to 50mM HEPES, pH 7.0. Fluorescein labeled Venus* and TEV protease, both containing a His tag, were bound to the HisTrap HP column. In order to perform biotinylation, 5mM TCEP (final concentration) and 25-fold molar excess biotinylation reagent were added to A2-77p*/A2-77p(AA)*. The biotinylation reagents contained either 2 PEG units (Maleimide-PEG_2_-Biotin, Pierce) or 77 PEG repeats (Maleimide-PEG_77_-Biotin, MW 3,400, Laysan Bio, Arab, AL). The biotinylated reaction was performed for 90min. at room temperature, followed by overnight incubation at 4°C. Reaction products A2-77p-short/A2-77p(AA)-short (containing 2 PEG-repeats) and A2-77p-long/A2-77p(AA)-long (containing 77 PEG-repeats) were separated from free biotinylation reagent using either P-2 fine gel resin (Bio-Rad Laboratories) or Zeba desalting column (7 MWCO, Thermo-Pierce).

### Gel electrophoresis

In some cases, small amounts of sample at every stage above were collected and resolved on 4–20% gradient SDS-PAGE gel under either reducing or non-reducing conditions. In other cases, 16% tricine gels with 6M urea were used to separate low molecular weight peptides under reducing conditions [21]. Tricine gels were run at 30V for 30min, 90V for 150min and then at 150V for 30min. Gel analysis involved either direct fluorescence measurement in gels by visualization under 254nm UV illumination, silver staining of proteins using a kit from Thermo-Pierce, or western blot analysis on nitrocellulose membranes using goat anti-biotin HRP conjugated antibody (Cell Signaling, Beverly, MA).

### Flow cytometry proteolysis assays

Biotinylated peptide substrates, A2-77p-short, A2-77p(AA)-short, A2-77p-long or A2-77p(AA)-long, were immobilized on 5μm streptavidin coated microspheres (Spherotech, Libertyville, IL, USA) by incubating them in PBS containing 1% bovine serum albumin (BSA) for 30min at room temperature. Typical proteolysis experiments proceeded in 20–50μl volume with ~400,000 microspheres being incubated with various concentrations/sources of ADAMTS13 in 50mM Tris buffer, pH 8.0 (final concentration). 2–5μl sample was withdrawn at fixed times, diluted in 200μl PBS and the microsphere associated fluorescein signal was measured using a FACScalibur flow cytometer (BD Biosciences, San Jose, CA, USA). Mean fluorescence intensity (MFI) was recorded and the extent of reaction was quantified. Initial reaction velocity (/min) was then calculated from the initial slope of the extent of reaction versus time plot.

In one study, bilirubin concentration was varied from 0–400 μM in the reaction buffer that contained 25% normal plasma ADAMTS13 activity. Here, a 2mM bilirubin (Sigma, St. Louis, MO, USA) stock solution was prepared in 0.1N NaOH, pH 8.0. This was mixed with HIP in different ratios to give bilirubin concentrations ranging from 2–1600μM. These samples were then mixed in a 1:1v/v ratio with pooled plasma from three normal donors, resulting in bilirubin from 1–800μM. These samples were then further diluted 1:1v/v in cleavage buffer containing A2-77p-long coated streptavidin microspheres. Thus, the final ADAMTS13 activity in all runs was ~0.25U/ml and bilirubin varied from 0–400μM.

### FRET studies

FRET based detection of ADAMTS13 activity was performed using the substrate XS-VWF as described previously [14], in 96-well microtiter plates using a BioTek Synergy 4 plate reader (Winooski, VT, USA) to monitor substrate cleavage. Here, following excitation at 420nm, FRET ratio quantifies ‘Light emission at 485nm/ Emission at 540nm’. FRET ratio increases upon XS-VWF proteolysis since the energy transfer from Cerulean (donor, ex: 433, em: 475) to Venus (acceptor, ex: 515 nm, em: 528 nm) decreases following proteolysis.

### Statistics

Data are presented as mean ± SEM for >3 independent experiments, unless otherwise mentioned. Paired t-test was applied for dual comparisons. *P*<0.05 was considered to be statistically significant.

## Results

### Preparation of ADAMTS13 substrate by N-terminal fluorescein labeling and C-terminal biotinylation

Fig 1A presents the detailed protocol used to create a 77-amino acid flow cytometry substrate to measure ADAMTS13 activity. Since the expression of this fragment alone in *E*. *coli* was not possible due to product instability, this peptide was expressed as a fusion protein with Venus (S1 Fig). Protein yield was ~3.5g/L of bacterial culture. The resulting *E*. *coli* derived 37.3 kDa protein A2-77p-Venus was fluorescent in SDS-PAGE gels when illuminated with 254nm UV light under non-reducing conditions (Fig 1D, lane 1). This signal was lost upon denaturing Venus by boiling the construct under reducing conditions (Fig 1B, lane 1). Fluorescein labeling of protein substrate to create A2-77p-Venus* resulted in bright fluorescence signal under both reducing (Fig 1B, lane 2) and non-reducing conditions (Fig 1D, lane 2), suggesting that the denaturing gel could be used to follow fluorescein labeling independent of Venus. Cleavage of A2-77p-Venus by TEV protease resulted in the release of fluorescein labeled Venus (Venus*, 27.5kDa) from the substrate of interest A2-77p* (9.8kDa, arrow), which was also fluorescein labeled (Fig 1B, lane 3, 4). A HisTrap HP column was used to isolate A2-77p* from Venus* and TEV protease since the latter molecules are his-tagged (Fig 1B, lane 5). The silver stain confirmed the purity of A2-77p* (Fig 1C, lane 4, arrow). In the last step, maleimide-(PEG)_n_-biotin was added in order to label the cysteines located near the C-terminus of A2-77p*. This final substrate with N-terminal fluorescein and C-terminal biotin is called A2-77p-short if there are two PEG units in the spacer and A2-77p-long when the spacer unit has 77 PEG units. Specific conjugation of fluorescein to the N-terminus and biotinylation of C-terminal segment is evident in the low-molecular weight tricine gels (Fig 1E). Here, the proteolysis of the 9.8kDa peptide A2-77p-short by recombinant ADAMTS13 (rADAMTS13) resulted in a fluorescent 1.5 kDa band (Fig 1E, top) and a biotinylated 8.3 kDa band (Fig 1E, bottom). Two additional molecules (A2-77p(AA)-short and A2-77p(AA)-long) where alanine replaced Y1606 and M1605 in either the short or long version of the substrate were created using this same protocol. These served as negative controls that were not cleavable by ADAMTS13. Overall, the above steps represent a systematic workflow for the creation of flow cytometry-microsphere substrates using *E*. *Coli*. The final peptide yield from the above steps was ~0.3g/L bacterial culture.

### A2-77p-short displays calcium-dependent ADAMTS13 mediated proteolysis

**Fig 2:**
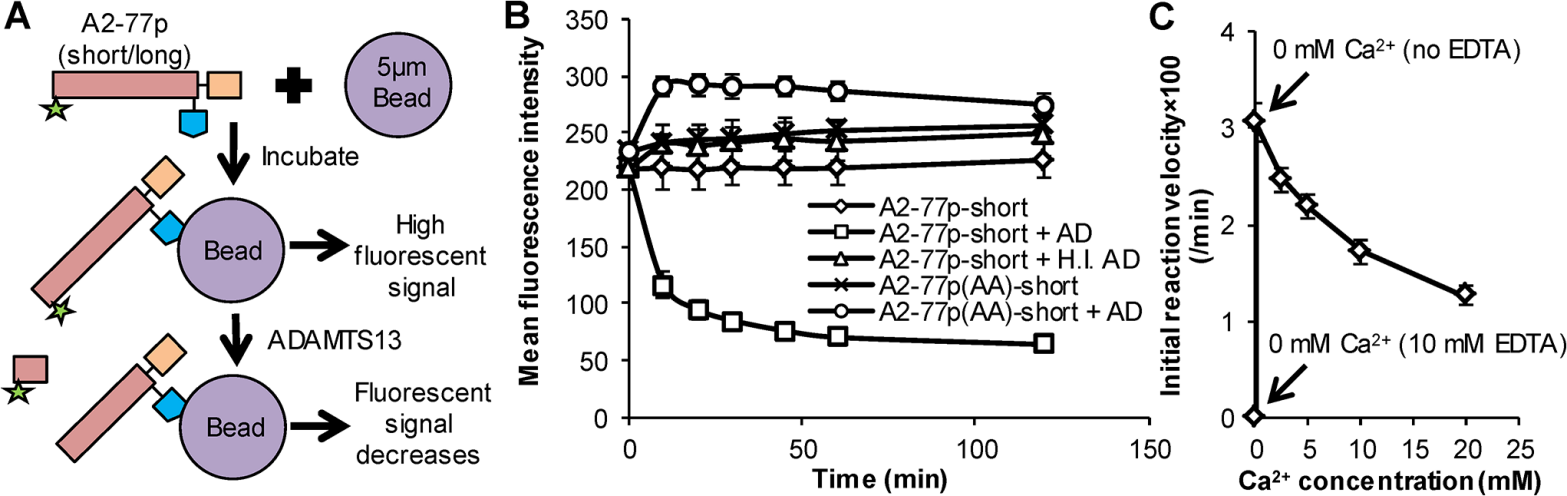
Specificity of the proteolysis assay and effect of calcium. **A.** Fluorescent, biotinylated substrate was immobilized on streptavidin coated microspheres. N-terminal proteolysis by ADAMTS13 reduced microsphere fluorescence as detected using flow cytometry. **B.** Cleavage of A2-77p-short was detected upon addition of 4.4 U/mL rADAMTS13 (AD). Microsphere fluorescence was unchanged when either rADAMTS13 was absent, rADAMTS13 was heat inactivated (H.I. AD), or when alanine mutated substrate A2-77p(AA)-short was used. Reaction buffer is 50mM Tris pH8.0, 0mM calcium. Error bars are too small to see in some cases. **C.** Cleavage of A2-77p-short by 0.55U/ml rADAMTS13 increased upon decreasing buffer calcium concentration. 10 mM EDTA in buffer abrogated substrate cleavage due to ADAMTS13 inactivation.

A flow cytometry based proteolysis assay was developed by incubating biotinylated A2-77p peptide variants with streptavidin bearing microspheres (Fig 2A). Due to the high affinity of the biotin-streptavidin bond, microspheres thus produced were stable for several weeks. Distinct microsphere fluorescein signal (MFI~225) was detected in the flow cytometer at *t* = 0s (Fig 2B). Addition of 4.4U/ml rADAMTS13 reduced this signal to MFI~50 in 1-2h in the case of A2-77p-short, with 80% of the signal decrease occurring within 10 min. The signal loss was due to the specific cleavage of the Y1605-M1606 bond by rADAMTS13 since the fluorescence signal did not change in the absence of rADAMTS13, when this protease was heat inactivated, or when the cleavage site was mutated to alanines (Fig 2B).

ADAMTS13 mediated proteolysis could be tuned by varying calcium concentration in the reaction buffer (Fig 2C). Whereas, decreasing calcium from 20mM down to 0mM increased proteolysis rates three-fold based on initial velocity estimates, addition of 10mM EDTA abrogated cleavage. The increased proteolysis at low calcium is consistent with a recent crystal structure which shows that calcium binding near the C-terminal α_3_β_4_ loop of VWF-A2 stabilizes the domain and reduced ADAMTS13 mediated cleavage [22]. The addition of EDTA abrogates cleavage since trace amounts of divalent ion (~60–80μM) are necessary for metalloprotease ADAMTS13 activity [23]. Based on the above, calcium was not added in all subsequent assays that measured ADAMTS13 activity (0mM Ca^2+^, no EDTA), unless otherwise mentioned.

### Detection of low levels of ADAMTS13 activity

**Fig 3:**
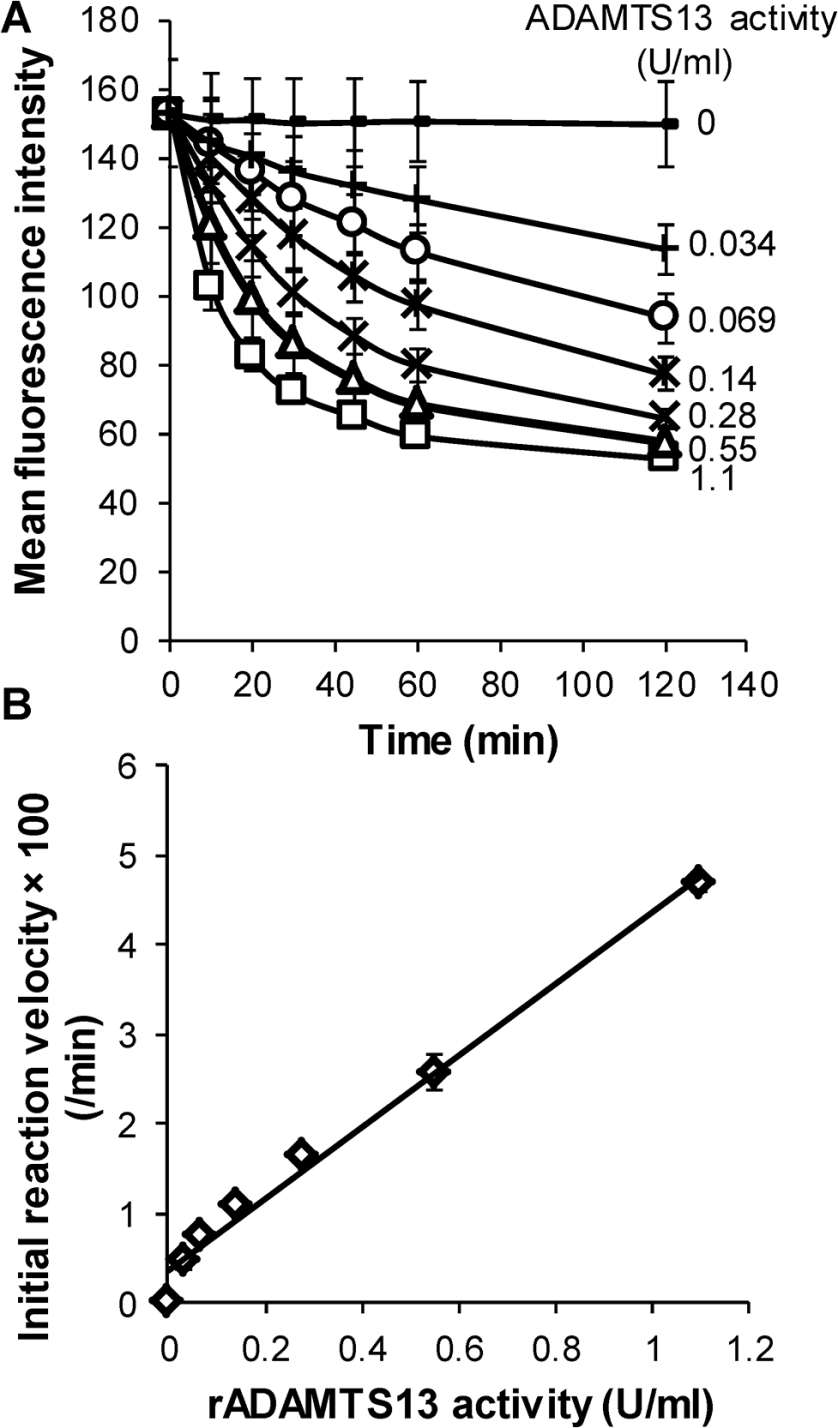
Dose-dependent proteolysis by rADAMTS13. **A.** Streptavidin coated microspheres bearing A2-77p-short were incubated with varying concentrations of rADAMTS13. Proteolysis was detected within 30min. even when protease concentration was 3.4% of normal plasma levels. **B.** Rate of proteolysis varied linearly with rADAMTS13 activity (R^2^ = 0.99).

In clinical settings, severe TTP diagnosis requires reliable measurement of low levels of ADAMTS13 activity <10% (i.e. <0.1U/ml) [24]. Thus, titration experiments were performed with rADAMTS13 to determine the sensitivity and linearity of the A2-77p-short substrate (Fig 3A). Here, initial reaction velocity was quantified based on proteolysis rates at early times when <50% cleavage had occurred. As seen, rADAMTS13 activity could be readily measured at 0.034U/ml, the lowest enzyme concentration tested, within 30min. Additionally, proteolysis rates varied linearly with rADAMTS13 activity (Fig 3B).

Lineweaver-Burk plots were generated by titrating the amount of A2-77p-short on the microspheres (S2 Fig). The K_M_ and V_max_ were calculated to be 219±15MFI and 3.61±0.24MFI/min, respectively. The MFI of 219 in our assays is achieved by incubating ~20ng of A2-77p-short (~9.8kDa) with ~400,000 microspheres for 30min. The washed microspheres were then resuspended in 20μl (proteolysis reaction volume). Hence, a K_M_ of 219MFI roughly translates to 0.1μM, assuming all of A2-77p-short was adsorbed by microspheres. This K_M_ is an order of magnitude lower than that reported for FRETS-VWF73 (3.2μM) [23] and XS-VWF (4.6μM) [14].

### Longer PEG spacer is necessary for substrate proteolysis by ADAMTS13 in plasma

**Fig 4:**
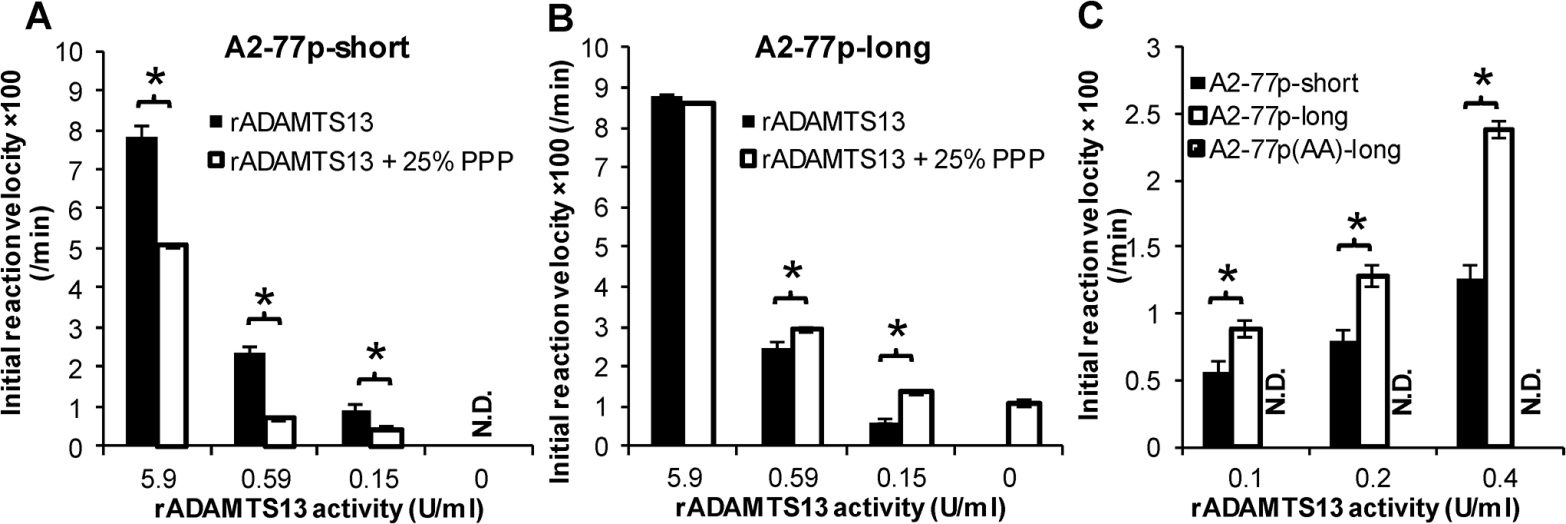
Effect of spacer length on proteolysis. **A.** A2-77p-short microspheres were incubated with varying concentrations of rADAMTS13 in the presence or absence of 25% plasma. Plasma failed to cleave A2-77p-short and also inhibited proteolysis of A2-77p-short by rADAMTS13. **B.** Experiments identical to panel A were performed, only using A2-77p-long. Here, plasma acts in synergy with rADAMTS13 to promote substrate cleavage. **C.** Direct comparison of the cleavage rates between A2-77p-short and A2-77p-long upon varying rADAMTS13 concentration. N.D. = cleavage not detected. *P<0.05 for indicated comparisons.

The ability of the A2-77p based substrates to measure ADAMTS13 in plasma was assayed (Fig 4). In studies using A2-77p-short, substrate proteolysis was not detected upon addition of 25% plasma (Fig 4A). Further, plasma reduced the rate of A2-77p-short cleavage in runs where additional rADAMTS13 was added. This observation was consistent regardless of donor or anti-coagulant: heparin, sodium citrate or citrate-dextrose. Changing the pH of the cleavage buffer from 8.0 to 6.0 also did not affect this finding.

We hypothesized that plasma components in the above experiments may sterically inhibit proteolysis by rADAMTS13. Further, such inhibition may be reduced by extending the substrate away from the microsphere surface. To test this, we developed A2-77p-long which contains the 77 unit PEG linker since this modification increases the extended PEG linker length from 0.7nm for A2-77p-short to 25nm for A2-77p-long [25]. Indeed, consistent with the above hypothesis, increasing PEG linker length in A2-77p-long allowed efficient substrate proteolysis by ADAMTS13 in plasma. Also, plasma acted in synergy with rADAMTS13 to promote cleavage (Fig 4B). Finally, the extent of enhancement noted here was consistent with the expected 0.25U/ml ADAMTS13 activity that would be expected from the 25% plasma used in the assay.

Finally, in studies that directly compared the cleavage of A2-77p-short vs. A2-77p-long at varying rADAMTS13 concentrations, a more rapid cleavage was noted for the substrate with the longer spacer unit (Fig 4C). No cleavage was detected for the negative control, A2-77p(AA)-long. Overall, a spacer length of 77 PEG units was sufficient to assay proteolysis when the reaction mixture contained 25% plasma.

### A2-77p-long proteolysis varies linearly with ADAMTS13 concentration in the presence of plasma

**Fig 5:**
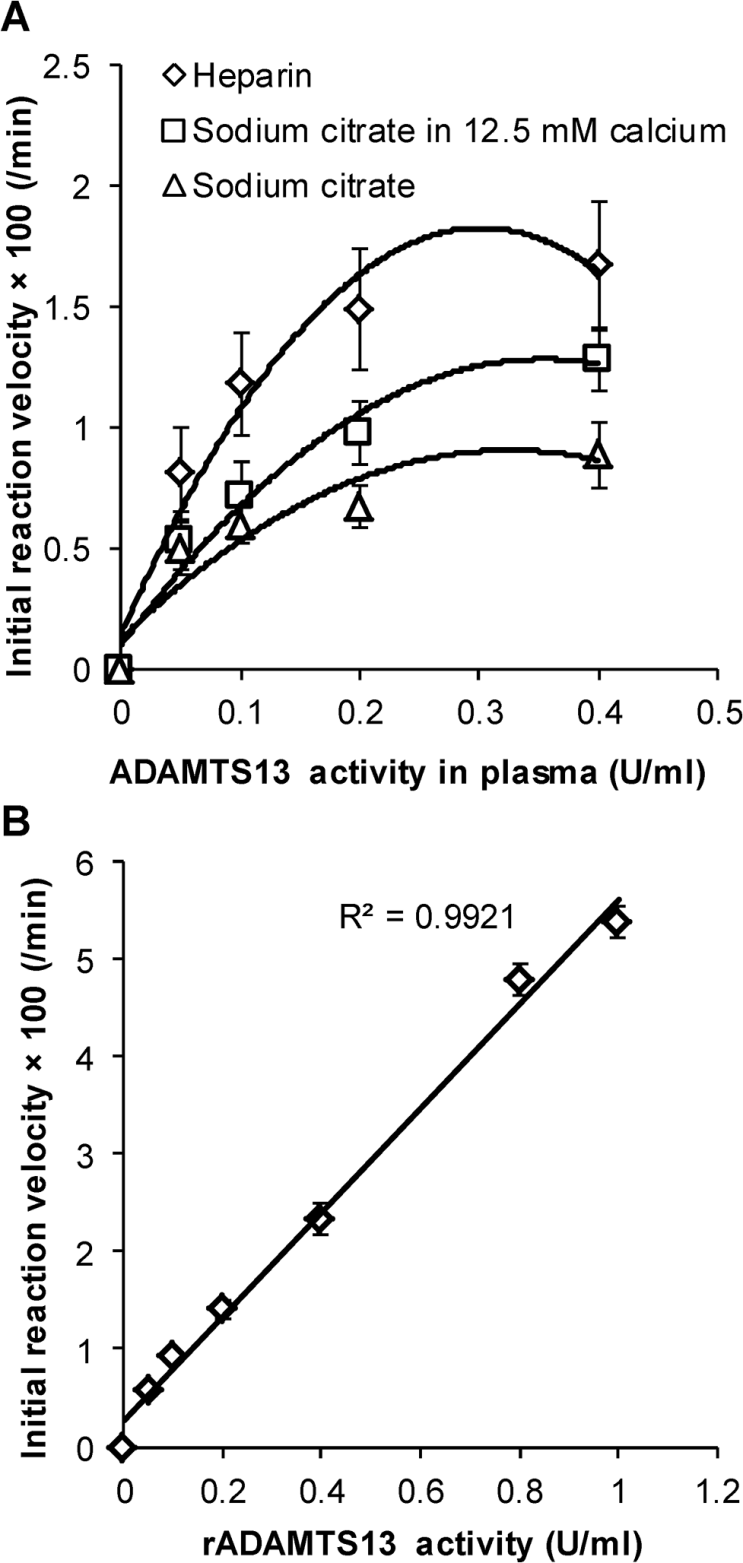
Dose-dependent proteolysis in runs containing plasma. **A.** Plasma was prepared from blood drawn using heparin or sodium citrate as anti-coagulant. This was diluted at indicated concentrations, into either normal Tris cleavage buffer or Tris cleavage buffer containing 12.5mM CaCl_2_. Microspheres bearing A2-77p-long were added. At all plasma dilutions, the cleavage reaction was maximum when using heparin as anti-coagulant. **B.** Microspheres bearing A2-77p-long were incubated with 25% HIP prepared in sodium citrate and 12.5 mM calcium. Recombinant ADAMTS13 was titrated at indicated concentrations. Proteolysis varied linearly with rADAMTS13 activity (R^2^ = 0.99).

Proteolysis experiments with A2-77p-long could be performed using plasma collected in either heparin or sodium citrate (Fig 5A). Here, ADAMTS13 mediated proteolysis proceeded more efficiently in heparin compared to sodium citrate, presumably because citrate reduced calcium levels below the ~60–80μM level that is required for ADAMTS13 activity. Consistent with this, the recalcification of citrated plasma with 12.5mM CaCl_2_ increased proteolysis kinetics. While substrate proteolysis generally increased with plasma concentration, the relationship was not strictly linear since many parameters changed simultaneously during these experiments including % plasma dilution and solution calcium concentration. Nevertheless, the data demonstrate that A2-77p-long and its analogs can provide functional readouts under a variety of conditions when plasma is obtained in different anti-coagulants.

In order to determine if substrate proteolysis varied linearly with ADAMTS13 activity in the presence of plasma, the A2-77p-long microspheres were mixed with 25% HIP (heat inactivated plasma) prepared in sodium citrate with 12.5mM calcium. Recombinant ADAMTS13 was then titrated from 0-1U/ml (Fig 5B). Here, proteolysis varied linearly with rADAMTS13 activity and enzyme activities down to 0.05U/ml were readily detected. Thus, the microsphere-cytometry based approach is optimal for plasma protease activity determination.

### Flow cytometry based assay is suitable even when plasma contains bilirubin

**Fig 6:**
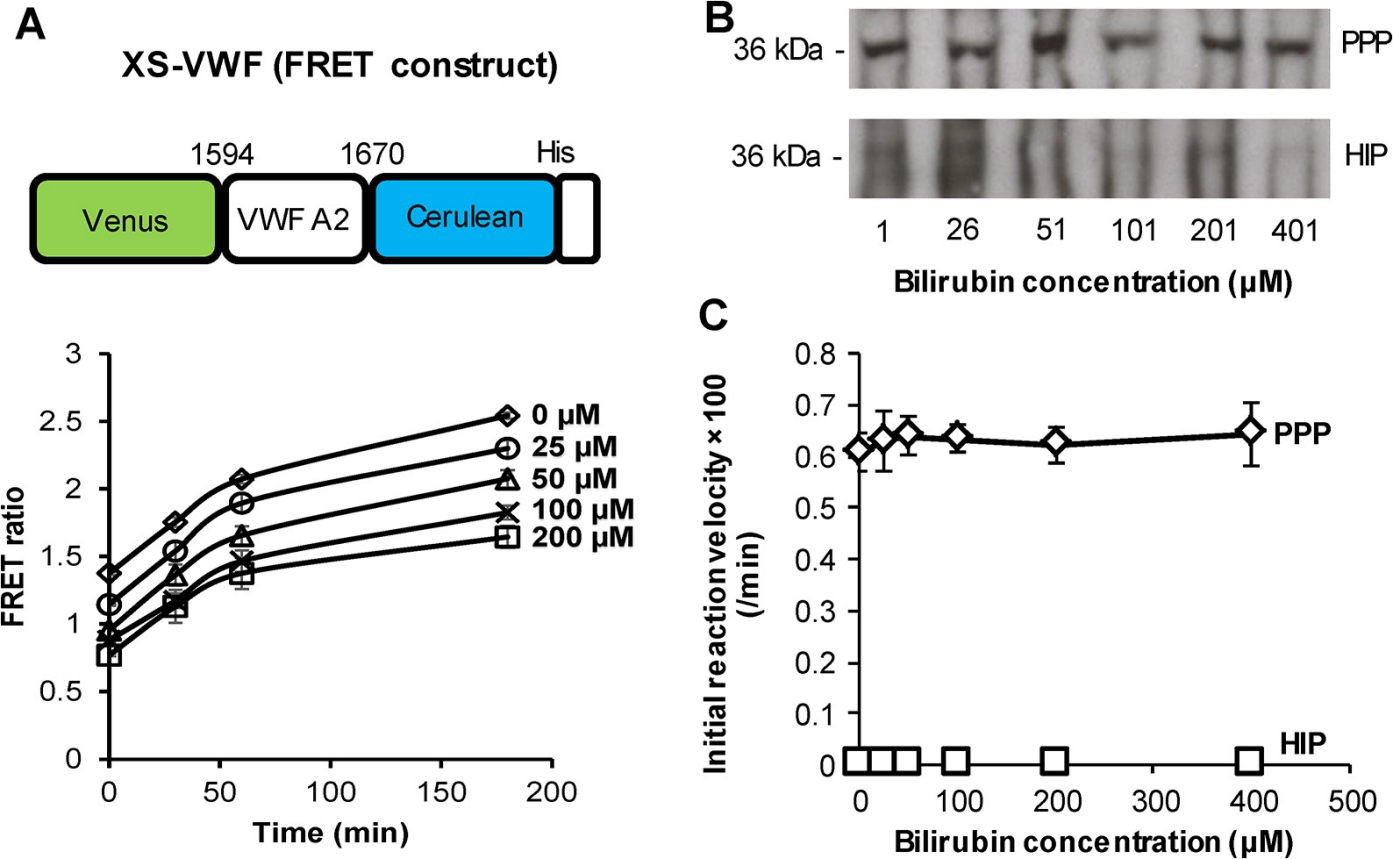
Effect of bilirubin on measurements of ADAMTS13 activity using XS-VWF and A2-77p-long. **A.** XS-VWF consists of Venus and Cerulean flanking the VWF-A2 77 amino acid peptide (residues 1594–1670). Substrate FRET ratio increases upon proteolysis. Here, 0.5μM XS-VWF was incubated with 25% normal human plasma containing different concentrations of recombinant bilirubin (0–400 μM). Baseline FRET ratio at *t* = 0s decreased upon increasing bilirubin dose. **B.** XS-VWF samples obtained at 60min. in panel A were analyzed using western blots and anti-tetra His antibody to detect the ~36kDa substrate cleavage band. Equal substrate proteolysis was detected independent of bilirubin in plasma (top). No proteolysis occurred in runs with HIP (bottom). **C.** A2-77p-long microspheres were incubated with 25% plasma (or 25% HIP) at different bilirubin concentrations (0–400μM). Substrate cleavage measured using flow cytometry was unaffected by bilirubin.

To further demonstrate the utility of the microsphere-cytometry assay, we compared measurements of ADAMTS13 activity using two substrates in the presence of bilirubin: the solution based XS-VWF FRET substrate (Fig 6A) versus immobilized A2-77p-long (Fig 6C). In the first case, an increase in XS-VWF FRET ratio was apparent upon addition of 25% normal human plasma, regardless of the solution bilirubin concentration. However, the baseline FRET ratio at *t* = 0 decreased upon increasing bilirubin. This was not due to differences in the extent of XS-VWF substrate cleavage upon bilirubin addition since western blotting shows equal intensity XS-VWF cleavage bands (~36 kDa) at all concentrations (Fig 6B). In this regard, the absorbance and emission spectra of bilirubin overlap with XS-VWF in the 400-500nm range, particularly with respect to Cerulean (S3 Fig). Due to this, both Cerulean excitation at 420nm and emission at 485nm are reduced by bilirubin and this diminishes the baseline FRET ratio. Regardless of this, XS-VWF can detect ADAMTS13 activity provided the data are normalized for baseline bilirubin correction.

In contrast to the solution based assays, the readout from A2-77p-long was identical regardless of the presence of bilirubin up to the highest concentration tested (400μM) (Fig 6C). Overall, the microsphere-cytometry assay performed better than the solution based FRET assays in the presence of contaminating bilirubin.

## Discussion

This study describes a novel *E*. *coli* derived substrate that can be applied in microsphere-cytometry assays to detect protease activity in human blood plasma. The advantage of this approach is that it requires 80-fold less reagent per assay compared to conventional fluorescence based solution assays [14]. Whereas the creation of a long 77 amino-acid substrate by chemical peptide synthesis is complicated, the availability of appropriate *E*. *coli* constructs streamlines substrate synthesis. Protein expression with a fused Venus also allows the colorimetric detection of the substrate during the isolation and chemical conjugation steps, and also enhances product solubility and yield. Inclusion of a single Lys residue, containing an ε-amino group, at the N-terminus allows specific fluorescence conjugation at this position. The fluorescence signal associated with the microspheres was stable and the reagent could be stored at 4°C for several weeks, the longest duration tested. Immobilization of the A2-77p substrate on microspheres allowed the rapid detection of clinically significant ADAMTS13 activity (<0.1U/mL) within 30 min since fluorescence associated with the microspheres decreased upon proteolysis. The substrate may also be adapted for high-throughput assays as the Z-factor is 0.72 for both A2-77p-short and A2-77p-long. Importantly, the introduction of the PEG spacer enabled the robust measurement of proteolytic activity when human blood plasma was directly added to the microspheres. Finally, the A2-77p substrate proteolysis readout was completely unaffected by fluorescent bilirubin, a metabolite that is often abnormally high in TTP patient plasma [15–17]. In this regard, the previously developed FRETS-VWF73 assay failed in the presence of bilirubin due to overlapping absorbance/fluorescence spectra [19]. For the same reason, the XS-VWF substrate also required background subtraction to account for baseline bilirubin fluorescence (Fig 6). With regard to this last aspect, while ELISA based methods allow the measurements of ADAMTS13 enzyme activity in clinical settings in the presence of bilirubin [24], the current study presents the first flow cytometry based assay for ADAMTS13 detection.

The measurement of ADAMTS13 antigen levels and enzyme activity is important for TTP diagnosis, and to distinguish the clinical symptoms of this disease from other thrombotic microangiopathies like Hemolytic-Uremic Syndrome (HUS), disseminated intravascular coagulation (DIC) and the HELLP syndrome [4,24]. Additionally, it is valuable to monitor this enzyme activity during von Willebrand Disease (VWD) type 2A since point mutations in the VWF A2-domain during this disease enhance VWF susceptibility to ADAMTS13-mediated proteolysis. This then leads to a loss of the larger VWF multimers and bleeding diathesis. While the substrate probe developed in the current work has advantages, it also suffers from the two limitations of the original substrate developed by Kokami [11,13] and derivatives developed by others (reviewed in [24]). First, under physiological conditions the proteolysis of VWF by ADAMTS13 is tightly regulated by fluid shear in that VWF exposed to hydrodynamic shear either in solution [26,27], when bound to platelets [28], or when immobilized on the endothelium [29] undergoes conformation changes that expose the cryptic A2-domain bond thus enabling mechanoenzymatic proteolysis. Fluid shear is absent in the current assay, and this is a similar limitation for other currently available ADAMTS13 diagnostic methods. Second, besides binding the VWF-A2 domain, the TSP5-CUB2 domains of ADAMTS13 also interact with the VWF D4-CK domains. In this regard, A2-77p-short/long and also other previously developed substrates contain only the C-terminal region of VWF-A2 and they lack additional VWF domains. Thus, defects in ADAMTS13 activity caused by mutations in TSP5-CUB2 domains will not be identified by these substrates. Additionally, while not related to this specific substrate, previous studies have shown that both thrombin and plasmin present in plasma can cleave ADAMTS13 and reduce its enzyme activity [3,4]. This in an important consideration when measurements of ADAMTS13 are performed using A2-77p and other related substrates.

The approach described in this work may be expanded to create a family of substrates that can assay additional enzyme activities in plasma in a multiplex manner. In this regard, the VWF A2-domain cleavage site in the current substrate is only 12 amino acids from the N-terminus. Thus, Gibson assembly or other generic molecular biology methods may be applied to the newly developed plasmid to clone a variety of sequences for other proteolytic enzyme sites in place of the current cleavage sequence. While this aspect has not been tested in the current work, the approach appears to be feasible given that a vast majority of substrates currently used to detect plasma protease activity are short peptides with few (i.e. 4–12) amino acids [12,30]. Additionally, given our knowledge of the spacer length necessary for efficient substrate cleavage in the milieu of plasma, it is now possible to develop simpler molecular constructs and methods to expand the scope of the current work in the future. For example, as an alternative to the PEG spacer, the introduction of Glycine/Serine based spacers into the plasmid DNA can allow defined control of the spacer arm that links the substrate to the microsphere [31]. Additionally, biotin can be directly incorporated *in situ* by engineering a peptide substrate for biotin-protein ligase at the C-terminus of the molecular construct [32]. Finally, a rainbow of fluorescence reporters can be conjugated to the N-terminal Lys for different protease substrates and their respective controls. Together, these modifications may not only enable the quantitation of specific protease activities in complex biological mixtures like blood plasma, they may also facilitate the development of new tools for high-throughput molecular screening and multiplex assays.

## Supporting Information

S1 Methods Supplemental Methods. (PDF)

S1 Table Cloning primers. (PDF)

S1 Fig Tricine gel followed by silver stain compares the expression of the peptide substrate expressed alone (left) or as a Venus fusion protein (right). Both molecules were purified using his-trap columns. Multiple bands and low expression was noted when the peptide substrate A2-77p-TEV-His was expressed alone suggesting protein instability (left lane). The substrate was however stably purified and functionalized when expressed as a fusion protein with Venus (A2-77p*, right lane). The peptide band appears at ~10 kDa (arrow). The peptide band in the right lane appears a little higher than the one in the left lane because it is labeled with fluorescein.
(TIF)

S2 Fig Lineweaver-Burk plot for the cleavage of A2-77p-short by rADAMTS13. Plot generated for runs where different amounts of A2-77p-short were immobilized on streptavidin coated microspheres, with rADAMTS13 being held constant at 0.55U/ml for all runs.
(TIF)

S3 Fig Effect of bilirubin on the absolute fluorescence (AFU) in the Cerulean and Venus channels. Fluorescence is measured in two channels for the FRET assay: Ch.1: Ex420nm/ Em485nm; and Ch.2:Ex420nm/Em540nm. Bilirubin (Bil) augments fluorescence in Ch.1 due to its spectral properties and depresses this in Ch.2 due to absorbance in this range. These two features affect XS-VWF FRET ratio. Figure shows fluorescence in Ch.1 and Ch.2 for PPP (panel **A**) and HIP (panel **B**) at 60min upon titrating bilirubin in the presence or absence of XS-VWF.
(TIF)
