# Supplementary material for: Detection of Plasma Protease Activity Using Microsphere-Cytometry Assays with E. coli Derived Substrates: VWF Proteolysis by ADAMTS13
Source: PLoS One. 2015 May 18;10(5):e0126556. doi: 10.1371/journal.pone.0126556 (PMC4436310; doi:10.1371/journal.pone.0126556)
Supplement: S1 Methods — (PDF) [file pone.0126556.s001.pdf]

Supplementary Methods

Protein expression and purification: The protocol is similar to one described previously (1). Briefly, all the proteins were expressed in the *E. coli* strain BL21 Star (DE3) pLysS (Invitrogen, Carlsbad, CA, USA). A single colony of *E. coli* bearing the required plasmid was inoculated in 1ml of Luria-Bertani (LB) broth containing 50μg/ml ampicillin. This was subsequently scaled up to 1L of culture. When the OD600 of the culture reached 0.6-0.7, protein expression was induced with 1 mM isopropyl b-D-thiogalactopyranoside (IPTG) (Sigma, St. Louis, MO, USA) for 12 h at 25°C. Cells were then centrifuged at 4000 rpm for 15 min, washed with phosphate-buffered saline, and resuspended in 10ml of 20mM HEPES buffer (pH 7.4) containing 300 mM NaCl, 10 mM imidazole, and ethylenediaminetetraacetic acid (EDTA)-free protease inhibitor cocktail tablets (Roche Diagnostics, Indianapolis, IN, USA). Lysozyme (final concentration 100μg/ml) was also added to facilitate cell lysis. Bacterial cells were lysed by three cycles of freeze–thaw. After this, the genomic DNA was sheared using a Branson sonifier (Danbury, CT, USA). Lysate was separated from the cell debris by centrifuging at 20,000g for 30 min, and subsequent filtration of the supernatant through a 0.22μm syringe filter. The supernatant was passed through a HisTrap HP column (GE Healthcare, Piscataway, NJ, USA) that was equilibrated with the same buffer used for cell lysis. The column was washed with 5-10 column volumes of 20mM HEPES buffer (pH 7.4) containing 300 mM NaCl and 58 mM imidazole. Protein was eluted with 20mM HEPES (pH 7.4) containing 300 mM NaCl and 250 mM imidazole.

TEV protease expression and purification: Plasmid pRK793 encoding for the catalytic domain of TEV protease, already in BL21 (DE3)-RIL, was purchased from Addgene (Cambridge, MA, USA). The cells were smeared onto LB agar plates containing 100μg/ml ampicillin and 30μg/ml chloramphenicol. After this step, the same protocol as in the previous section was followed to purify the enzyme except for a few changes: i. Ampicillin (final concentration 100μg/ml) and chloramphenicol (final concentration 30μg/ml)

were used in the LB culture. ii. Induction was carried out for 5h at 30°C. iii. The buffers used for HisTrap HP column contained 50mM NaH_2PO_4 (pH 8.0) instead of 20mM HEPES (pH 7.4).

References

1. Dayananda KM, Gogia S, Neelamegham S. Escherichia coli-derived von Willebrand factor-A2 domain fluorescence/Förster resonance energy transfer proteins that quantify ADAMTS13 activity. Analytical Biochemistry 2011; 410: 206-13.
