# Supplementary figures and images for: Detection of Plasma Protease Activity Using Microsphere-Cytometry Assays with E. coli Derived Substrates: VWF Proteolysis by ADAMTS13
Source: PLoS One. 2015 May 18;10(5):e0126556. doi: 10.1371/journal.pone.0126556 (PMC4436310; doi:10.1371/journal.pone.0126556)

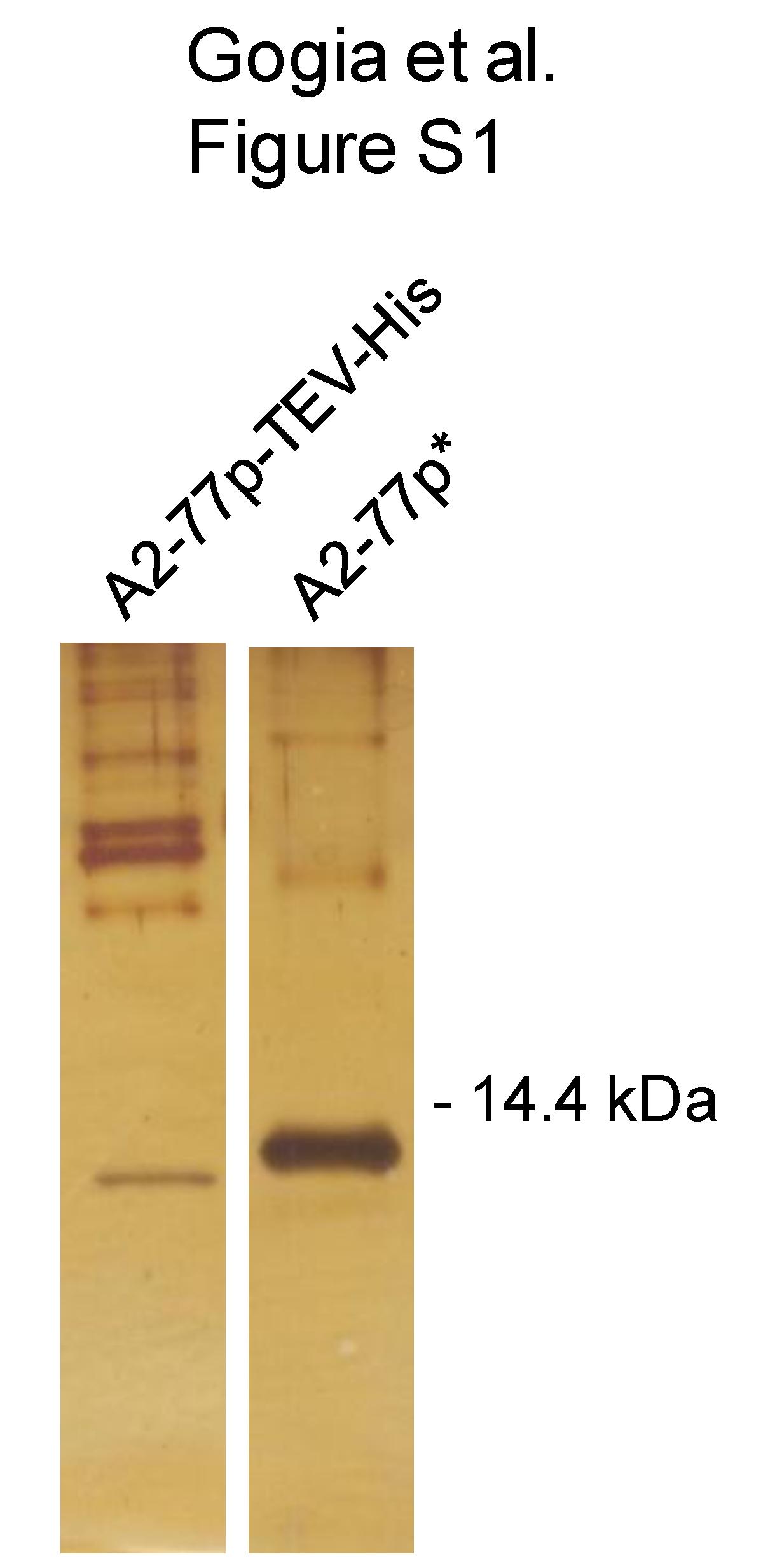

Supplement: S1 Fig — Both molecules were purified using his-trap columns. Multiple bands and low expression was noted when the peptide substrate A2-77p-TEV-His was expressed alone suggesting protein instability (left lane). The substrate was however stably purified and functionalized when expressed as a fusion protein with Venus (A2-77p*, right lane). The peptide band appears at ~10 kDa (arrow). The peptide band in the right lane appears a little higher than the one in the left lane because it is labeled with fluorescein. (TIF) [file pone.0126556.s003.tif]
